# Causes of virological failure in a population of 1895 HIV-infected patients: the experience of an infectious diseases service in Lisbon, Portugal

**DOI:** 10.7448/IAS.15.6.18065

**Published:** 2012-11-11

**Authors:** V Moneti, N Luís, J Rijo, A Miranda, T Baptista, H Farinha, K Mansinho

**Affiliations:** 1 Hospital Egas Moniz/Centro Hospitalar de Lisboa Ocidental E.P.E., Infectious Diseases and Tropical Medicine, Lisbon, Portugal; 2 Hospital Egas Moniz/Centro Hospitalar de Lisboa Ocidental E.P.E., Pharmacy, Lisbon, Portugal

## Abstract

Despite the increasing optimization of combined antiretroviral therapy (cART) regimens in the last decades, a significant percentage of patients still do not achieve viral replication control. We present a retrospective analysis focusing on human immunodeficiency virus (HIV)-infected population on cART, followed at our ambulatory care clinic between 1st January and 31st December 2011, in order to identify the causes of virological failure. From the 1895 patients in our population we included 1854 in the study. Ten percent (187) of the included patients had detectable HIV RNA (≥40 cp/mL) at the time of last laboratory evaluation: 70,1% were males, mean age was 46 years and 72,7% were Portuguese. Patients with detectable HIV RNA were divided into group A (HIV RNA <200 cp/mL) - 78 (41,7%) patients and group B (HIV RNA ≥200 cp/mL) 109 (58,3%) patients. The comparison of both groups revealed an higher mean count of TCD4+ (568 vs 334 cells/mm^3^; p<0,001) in group A, although similar mean TCD4+ count at time of cART initiation (276 vs 262 cells/mm^3^; p=0,412). Group A patients experienced longer exposure to cART (10 vs 8 years; p<0,05) and have undergone, on average, 3 previous regimens (p<0,05). With regard to cARV current regimen: 32,1% patients in group A and 30,3% in group B were prescribed non-nucleoside reverse transcriptase inhibitors based regimes and 51,3% patients in Group A and 59,6% in group B were under cARV based on Protease inhibitors. The identified causes of virologic failure for patients with detectable HIV RNA were: poor adherence (54%); unsuccessful retention in care (14,4%); sporadic detectable HIV RNA (40≤viral load<200), “blips” (14,4%); mutations of resistance to ARVs (13,4%); intolerance to the current regimen (2,1%) and pharmacokinetics drug interactions (1,6%). The estimated rate of virological failure was 10,1% in this population. Insufficient adherence and unsuccessful retention in care were identified in 68,4% of treatment failed patients as main causes of virological failure. Failure of therapy due to intolerance or adverse effects was reported in 2,1% of cases, reflecting a better safety profile and tolerability of recent prescribed regimens. Early identification of causes of virologic failure, timely adjustment of therapeutic regimens, and the adoption of measures to promote adherence and retention in care are key factors for successful treatment of HIV-infected patients.

